# Editorial: Designing metal-complexes (metallo-drugs) against infectious diseases and understanding their metal toxicity

**DOI:** 10.3389/fcimb.2024.1463451

**Published:** 2024-08-28

**Authors:** Sanjay Kumar Rohaun

**Affiliations:** Department of Microbiology, University of Illinois at Urbana-Champaign, Champaign, IL, United States

**Keywords:** drug resistance, nanoparticle, metal, microbe, antibiotics

Infectious diseases remain a formidable challenge in medicine, exacerbated by the rise of antibiotic resistance (de la Fuente-Nunez et al., 2023). The quest for innovative solutions has prompted the scientific community to explore new strategies for antimicrobial therapy. One promising avenue is the design and application of metal complexes (metallo-drugs), which utilize the unique properties of metal ions to enhance antimicrobial activity (Frei et al., 2023; Waters et al., 2023). These complexes have demonstrated significant antimicrobial potential and often possess unique modes of action that can overcome the resistance mechanisms employed by bacteria, fungi, and other pathogens. However, understanding and mitigating the inherent metal toxicity of these compounds is crucial for their safe and effective use. This editorial delves into the significant contributions of five research groups on metallo-drugs, focusing on their design, efficacy, mechanism of action, and toxicity. Recent research highlights the potential of these compounds, offering hope against resistant strains of bacteria and fungi. However, a critical understanding of metal toxicity is essential to ensure the safety and efficacy of these novel treatments.

## Platinum complexes against MRSA

Platinum complexes are well-known for their anticancer properties, but their antimicrobial potential is less explored. Methicillin-resistant *Staphylococcus aureus* (MRSA) represents a significant public health challenge due to its resistance to multiple antibiotics. Nam-Cha et al. have made significant strides in synthesizing and characterizing heteroscorpionate derivative platinum complex. This novel platinum complex exhibited potent antibacterial properties against MRSA, highlighting the potential of platinum-based drugs in combating resistant bacterial strains. The heteroscorpionate ligand’s ability to stabilize the platinum center and facilitate interactions with bacterial targets underscores the importance of ligand design in enhancing the efficacy of metal-based antimicrobials. The significance of this study lies in its potential to overcome MRSA’s resistance mechanisms, offering a potent alternative to traditional antibiotics. Their work demonstrated that the platinum complex disrupts bacterial cell walls and inhibits critical enzymatic processes.

## Iridium complexes in anti-fungal therapy

Fungal infections, particularly those caused by resistant strains of *Candida albicans*, pose a severe threat to immunocompromised patients. The study by Lu et al. demonstrated the anti-fungal use of cyclometalated iridium (III) and polypyridyl ruthenium(II) complexes in combination with fluconazole, a commonly used anti-fungal drug. The combination therapy exhibited synergistic effects, significantly enhancing anti-fungal activity against resistant *C. albicans*. Importantly, *Candida’s* yeast state to hyphal state morphological transformation of resistant was also inhibited and showed mitochondrial damage with ROS production. This combination approach improves efficacy and reduces the required dosage of fluconazole, potentially mitigating its side effects. This study exemplifies how metallo-drugs can be integrated with existing anti-fungals to tackle resistant fungal pathogens effectively.

## Palladium(II) complexes and efflux pump inhibition

Efflux pumps are a common resistance mechanism in bacteria, expelling antibiotics and reducing their intracellular concentrations. Shobana et al. explored the effect of palladium(II) complexes on the NorA efflux pump in fluoroquinolone-resistant *Staphylococcus aureus*. Their *in vitro* and in silico studies revealed that these palladium complexes in unique node effectively resensitizing the bacteria to fluoroquinolones. This dual approach of using metal complexes to inhibit resistance mechanisms and enhance antibiotic efficacy significantly advances the fight against resistant bacterial infections. The study also highlighted the importance of computational methods in understanding the interaction between metal complexes and bacterial proteins.

## Silver bio-functionality and implant-associated infections

For a long time, silver is known for its antimicrobial properties, but concerns over toxicity often limit its clinical use. Cecotto et al. evaluated the bio-functionality of silver in a sophisticated multicellular *in vitro* model to mimic the human cellular environment to address the challenges of implant-associated infections. Silver’s well-known antimicrobial properties were validated in a controlled environment that mimics human tissue, providing insights into its efficacy and safety. This multicellular model offers valuable insights into the biocompatibility and toxicity of metal complexes. It investigates host-bacteria and host-host interactions, including inflammatory responses. By using advanced *in vitro* models, this research paves the way for safer and more effective utilization of silver in medical applications, reducing reliance on animal testing and addressing toxicity concerns.

## Biosynthesis of multimetallic nanoparticles against ESBL-producing *E. coli*

The emergence of extended-spectrum beta-lactamase (ESBL) producing *Escherichia coli* in veterinary settings necessitates new antimicrobial strategies. Rasheed et al. investigated the antimicrobial potential of fungus-mediated synthesis of Se-BiO-CuO multimetallic nanoparticles (NP) targeting ESBL-producing *Escherichia coli* of veterinary origin. These extended-spectrum beta-lactamase (ESBL) producing bacteria are notorious for resisting conventional antibiotics, posing significant risks to animal and human health. The NP biosynthesis approach is environmentally friendly and cost-effective, leveraging fungal metabolism to produce nanoparticles with potent antimicrobial properties. These nanoparticles exhibited remarkable antibacterial activity, suggesting that the biogenic synthesis of multimetallic nanoparticles could serve as a viable alternative to traditional antibiotics. Incorporating selenium, bismuth, and copper oxide provides a multifaceted mechanism of action, enhancing their effectiveness against resistant bacteria.

While the antimicrobial potential of metal complexes is promising, their clinical application is often hampered by concerns over metal toxicity. Understanding and mitigating these toxic effects is crucial for the safe use of metallo-drugs. Strategies to address metal toxicity:

Ligand Design: Developing ligands that specifically target microbial cells while sparing human cells can reduce off-target effects and enhance the therapeutic index of metallo-drugs.

Nanoparticle Encapsulation: Encapsulating metal complexes in biocompatible nanoparticles can improve drug delivery, enhance targeting specificity, and reduce systemic toxicity.

*In Vitro* Models: Advanced *in vitro* models, such as the multicellular systems used by Cecotto et al. provide valuable insights into the biocompatibility and toxicity of metal complexes, facilitating safer design and application.

Combination Therapies: Combining metal complexes with existing antibiotics or anti-fungals can reduce the required dose of each component, minimizing potential toxicity while maximizing therapeutic efficacy. Additionally, Shobana et al. emphasize the use of in silico methods to predict and optimize the interactions of metal complexes with bacterial targets, reducing the likelihood of adverse effects.

The design and application of metal complexes hold significant promise in the fight against infectious diseases, particularly those caused by drug-resistant pathogens. The recent advancements in platinum, iridium, palladium, silver, and multimetallic nanoparticles underscore these compounds’ diverse and potent antimicrobial capabilities. As we continue to explore this exciting frontier, interdisciplinary research combining chemistry, microbiology, and pharmacology is essential to harness the full potential of metallo-drugs in the ongoing battle against infectious diseases. Metallo-drugs research holds promise for a new era in treating infectious diseases, potentially transforming the landscape of antimicrobial therapy. This topic will give exciting insights into therapeutic approaches to treating infectious diseases.
